# Mesenchymal stem cell therapy on top of triple therapy with remdesivir, dexamethasone, and tocilizumab improves PaO_2_/FiO_2_ in severe COVID-19 pneumonia

**DOI:** 10.3389/fmed.2022.1001979

**Published:** 2022-09-23

**Authors:** Chih-Hao Chen, Kuan-Cheng Chang, Yen-Nien Lin, Mao-Wang Ho, Meng-Yu Cheng, Wen-Hsin Shih, Chia-Huei Chou, Po-Chang Lin, Chih-Yu Chi, Min-Chi Lu, Ni Tien, Mei-Yao Wu, Shih-Sheng Chang, Wu-Huei Hsu, Woei-Cheang Shyu, Der-Yang Cho, Long-Bin Jeng

**Affiliations:** ^1^Division of Infectious Diseases, Department of Internal Medicine, China Medical University Hospital, Taichung, Taiwan; ^2^Division of Cardiovascular Medicine, Department of Internal Medicine, China Medical University Hospital, Taichung, Taiwan; ^3^School of Medicine, China Medical University, Taichung, Taiwan; ^4^Department of Microbiology and Immunology, School of Medicine, China Medical University, Taichung, Taiwan; ^5^Department of Laboratory Medicine, China Medical University Hospital, Taichung, Taiwan; ^6^School of Post-Baccalaureate Chinese Medicine, China Medical University, Taichung, Taiwan; ^7^Department of Chinese Medicine, China Medical University Hospital, Taichung, Taiwan; ^8^Division of Pulmonary Medicine, Department of Internal Medicine, China Medical University Hospital, Taichung, Taiwan; ^9^Translational Medicine Research Center, China Medical University Hospital, Taichung, Taiwan; ^10^Department of Neurology, China Medical University Hospital, Taichung, Taiwan; ^11^Graduate Institute of Biomedical Sciences, China Medical University, Taichung, Taiwan; ^12^Stroke Center, China Medical University Hospital, Taichung, Taiwan; ^13^Department of Neurosurgery, China Medical University Hospital, Taichung, Taiwan; ^14^Organ Transplantation Center, China Medical University Hospital, Taichung, Taiwan

**Keywords:** arterial partial pressure of oxygen vs. fraction of inspired oxygen, COVID-19, human umbilical cord mesenchymal stem cells, monocyte distribution width, inflammatory cytokines

## Abstract

**Background:**

Despite patients with severe coronavirus disease (COVID-19) receiving standard triple therapy, including steroids, antiviral agents, and anticytokine therapy, health condition of certain patients continue to deteriorate. In Taiwan, the COVID-19 mortality has been high since the emergence of previous variants of this disease (such as alpha, beta, or delta). We aimed to evaluate whether adjunctive infusion of human umbilical cord mesenchymal stem cells (MSCs) (hUC-MSCs) on top of dexamethasone, remdesivir, and tocilizumab improves pulmonary oxygenation and suppresses inflammatory cytokines in patients with severe COVID-19.

**Methods:**

Hospitalized patients with severe or critical COVID-19 pneumonia under standard triple therapy were separated into adjuvant hUC-MSC and non-hUC-MSC groups to compare the changes in the arterial partial pressure of oxygen (PaO_2_)/fraction of inspired oxygen (FiO_2_) ratio and biological variables.

**Results:**

Four out of eight patients with severe or critical COVID-19 received either one (*n* = 2) or two (*n* = 2) doses of intravenous infusions of hUC-MSCs using a uniform cell dose of 1.0 × 10^8^. Both high-sensitivity C-reactive protein (hs-CRP) level and monocyte distribution width (MDW) were significantly reduced, with a reduction in the levels of interleukin (IL)-6, IL-13, IL-12p70 and vascular endothelial growth factor following hUC-MSC transplantation. The PaO_2_/FiO_2_ ratio increased from 83.68 (64.34–126.75) to 227.50 (185.25–237.50) and then 349.56 (293.03–367.92) within 7 days after hUC-MSC infusion (*P* < 0.001), while the change of PaO_2_/FiO_2_ ratio was insignificant in non-hUC-MSC patients (admission day: 165.00 [102.50–237.61]; day 3: 100.00 [72.00–232.68]; day 7: 250.00 [71.00–251.43], *P* = 0.923).

**Conclusion:**

Transplantation of hUC-MSCs as adjunctive therapy improves pulmonary oxygenation in patients with severe or critical COVID-19. The beneficial effects of hUC-MSCs were presumably mediated by the mitigation of inflammatory cytokines, characterized by the reduction in both hs-CRP and MDW.

## Introduction

Despite great efforts to control and eradicate the pandemic, the number of confirmed patients with severe acute respiratory syndrome corona virus 2 (SARS-CoV-2) infection has reached more than 568 million worldwide by July 23, 2022, with more than 6 million deaths (1). Although numerous potential approaches to prevent or treat novel coronavirus disease 2019 (COVID-19), such as vaccines, oligonucleotides, and monoclonal antibodies, have been applied in clinical use (2), one fifth of COVID-19 cases still result in serious symptoms (3, 4). In severely or critically ill patients, provocation of a systemic hyperimmune response may lead to prolonged respiratory distress syndrome and multiple end-organ dysfunctions with high mortality (5). Contemporary guideline-recommended management for patients with severe or critical disease includes the use of a high-flow nasal cannula (HFNC), standard triple therapy with remdesivir, dexamethasone, and tocilizumab, and supportive care (6). In addition to these regular treatments, therapies with acceptable safety profiles that are capable of decreasing or modulating inflammation may be beneficial at this stage (7).

Mesenchymal stem cells (MSCs) have drawn considerable attention because of their anti-inflammatory and immunomodulatory effects (8–10). Currently, MSCs are widely used in cell therapy and approved for the treatment of Crohn’s disease or graft-vs.-host disease (11, 12). The safety and effectiveness of MSCs have been documented in several clinical trials (11, 12). Among the various MSC types, human umbilical cord MSCs (hUC-MSCs) can be easily obtained and cultured. hUC-MSCs have shown immunomodulatory and tissue-repair capabilities with low immunogenicity, which makes them ideal candidates for allogeneic adoptive transfer therapy (13). The anti-inflammatory and immunomodulatory properties of MSC therapy merit consideration as a potential therapeutic strategy for COVID-19 (13, 14). To date, more than 60 clinical trials have investigated the safety and efficacy of adult allogeneic or autologous MSCs in patients with COVID-19. Among these, hUC-MSCs and adipose-derived MSCs are the most commonly used cell types.

Several studies have reported the clinical benefits of hUC-MSCs in diverse clinical settings of SARS-CoV-2 infection (15–18). Iglesias et al. (19) showed that the infusion of hUC-MSCs in five patients with severe acute respiratory distress syndrome (ARDS) caused by COVID-19 improved respiratory function, which was expressed by the ratio of arterial oxygen partial pressure (PaO_2_)/fraction of inspired oxygen (FiO_2_) (PaO_2_/FiO_2_) in three patients recovering from severe respiratory failure. However, whether cell therapy was added to the standard triple therapy consisting of steroid, antiviral, and anti-cytokine agents in these patients was not clear. This study was also limited by the lack of a control group for the comparison and comprehensive analysis of inflammatory cytokines. Recently, a double-blind, phase 1/2a randomized controlled trial was conducted to assess the safety and explore the efficacy of hUC-MSC infusions in patients with COVID-19 ARDS (16). The results of this study showed that inflammatory cytokines were significantly decreased in hUC-MSC-treated patients, with significantly improved patient survival. However, not all patients in the hUC-MSC group received the standard triple therapy. Notably, there was only 1 patient (8.3%) in the hUC-MSC group who received tocilizumab treatment. Another randomized controlled trial showed similar survival benefits in hUC-MSC-treated patients with COVID-19 (18). However, neither the treatment group nor the control group received standard triple therapy. In addition, the baseline disease severity of patients with COVID-19 was not well characterized in the study patients according to the definition by contemporary guidelines (6). Therefore, it remains unclear whether the infusion of hUC-MSCs exerts beneficial effects in addition to standard triple therapy with a combination of steroid, antiviral, and anticytokine agents in severe or critical patients with COVID-19.

During the emergence of previous variants of COVID-19 (such as alpha, beta, or delta), despite severe or critical patients with COVID-19 receiving guideline-directed management, health condition of certain patients continued to deteriorate, owing to which, COVID-19 mortality remains high in Taiwan (3.89% as of March 19, 2022) (20). Based on the beneficial effects of MSCs, we hypothesized that transplantation of hUC-MSCs, as a compassionate therapy, may be beneficial in addition to standard triple therapy in critical patients with COVID-19. Herein, we conducted a case-control study to describe the clinical characteristics, laboratory findings, changes in inflammatory cytokines, and outcomes of patients with severe or critical COVID-19 who received one or two doses of adjuvant hUC-MSC infusion in addition to dexamethasone, remdesivir, and tocilizumab treatment.

## Materials and methods

### Patients

Hospitalized patients who were diagnosed with COVID-19 by positive real-time polymerase chain reaction (RT-PCR) for SARS-CoV-2 from nasal swab specimens were enrolled in this retrospective case-control study between May 15, 2021 and June 15, 2021. The disease severity of patients with COVID-19 was classified as mild, moderate, severe, and critical according to the following criteria: (1) tachypnea (RR ≥ 30 times/min), (2) finger oxygen saturation ≥93% in the resting state, and (3) PaO_2_/FiO_2_ ≤ 300 mmHg, as determined by the National Institute of Health treatment guidelines (2). Patients with severe COVID-19 must fulfill all three criteria. If these patients develop septic shock or respiratory disease and have multiorgan dysfunction, they are considered to have a critical COVID-19 infection. Symptomatic and supportive treatments were initiated for patients with mild or moderate disease. Patients with severe or critical disease underwent standard management, including the use of a HFNC, standard triple therapy with remdesivir, dexamethasone, and tocilizumab, and supportive treatments according to the treatment guidelines (2). Endotracheal tube insertion and mechanical ventilation were initiated when patients developed acute respiratory failure during the HFNC treatment. Patients with severe or critical disease were candidates for adjuvant hUC-MSC transplantation in addition to standard treatment. Patients who received hUC-MSC transplantation agreed to this compassionate therapy, and the adjuvant hUC-MSC treatment was approved by the Taiwan Ministry of Health and Welfare on a case-by-case basis. Patients with severe or critical disease were then separated into hUC-MSC and non-hUC-MSC groups for comparison according to whether hUC-MSCs were administered. The local Institutional Review Board (CMUH-110-REC1-123) approved all data collection during the study period.

### Patient and public involvement statement

Owing to the retrospective nature of the analysis, it was not appropriate or possible to involve patients or the public in the design, conduct, reporting, or dissemination plans of our research.

### Preparation and characterization of human umbilical cord mesenchymal stem cells

The umbilical cord from an eligible donor who had consented to its use for hUC-MSC production was cut with a sterile scalpel to 1 cm in length after removing the blood vessels. The umbilical tissue was washed with Hank’s Balanced Salt Solution (Biological Industries, Kibbutz Beit Haemek, Israel) containing gentamicin (0.03 mg/mL; Yungshin Pharm Ind., Co., Ltd., Taipei, Taiwan) and fungizone (0.0025 mg/mL; Bristol-Myers Squibb, NY, United States), chopped into small pieces (∼1 mm^3^), and digested with collagenase type I (2 mL/cm cord length; Thermo Fisher Scientific Inc., MA, United States) in an incubator at 37°C for 2 h. Dulbecco’s phosphate-buffered saline was mixed with the umbilical extract, filtered with 100 μm cell strainers, and centrifuged; the final cell pellet was resuspended in growth medium (Biological Industries) with Cell Culture Supplement (HELIOS BioScience, Creteil, France) and gentamicin and then cultured in a CellBind-coated 25T Flask (Thermo Fisher Scientific, Inc.) in a 5% CO_2_ incubator at 37°C. Metabolic waste was removed and nutrition was replenished every 3–4 days by replacing the growth medium. When the cells reached 95% confluence, they were detached using TrypLE (Thermo Fisher Scientific, Inc.) and then seeded in a hyperflask at a density of 2000–4000 cells/cm^2^ in a 5% CO_2_ incubator at 37°C for 2–4 passages (approximately 6–8 weeks) for the final harvest. The cells were washed and detached with TrypLE, and the cell pellet was resuspended at a final cell density of ∼1.0 × 10^7^ cells/mL in a 1:1 ratio of CS10 (Biolife Solution Inc., WA, United States) and albumin (CSL Behring, PA, United States). The cell suspension was preserved in a liquid nitrogen cell tank for further clinical use.

**FIGURE 1:**
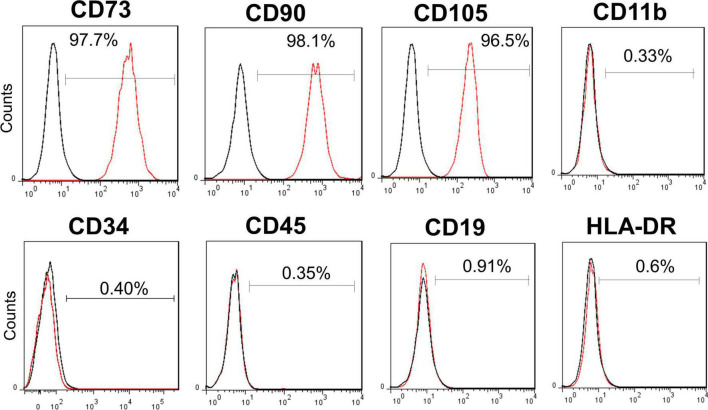
Surface markers of allogenic human umbilical cord mesenchymal stem cell (hUC-MSC). Flow cytometry analysis of hUC-MSC showing the positive surface markers CD73, CD90, CD105 (> 95%), and the negative markers CD11b, CD19, CD34, CD45, and HLA-DR (0.33%, 0.91%, 0.40%, 0.35%, and 0.6%, respectively). CD, cluster of differentiation; HLA-DR, major histocompatibility complex (MHC) II cell surface receptor of human leukocyte antigen.

On the day of administration, the cryopreserved cells were thawed in a 37°C water bath. The cell suspension was washed thrice to minimize residual reagents during the manufacturing process. The cell pellet was resuspended in normal saline at a final cell density of ∼1 × 10^7^ cells/mL for administration. The cell identity test showed that ≥ 95% of cells expressed CD73, CD90, and CD105, while the expression levels of CD11b, CD19, CD45, CD34, and HLA-DR were ≤2% (Figure 1). The final volume of hUC-MSC suspension for infusion was 100 mL 0.9% saline containing a cell dose of 1.0 × 10^8^ and fulfilled the following product release criteria: cell viability by trypan blue (> 70%), purity test by endotoxin examination (<0.25 EU/mL), and sterility test by gram staining and direct inoculation (negative) (21).

### Measurement of inflammatory cytokines

To measure serial changes in inflammatory cytokines, chemokines, and growth factors in patients who received hUC-MSC transplantation, a multiplexed bead-based immunoassay (Cytometric Bead Array, Becton Dickinson, NJ, United States) was used to determine the plasma levels of these cytokines, including granulocyte-macrophage colony-stimulating factor (GM-CSF), interferon gamma (IFN-γ), interleukin (IL)-1β, IL-2, IL-4, IL-6, IL-8, IL-12, IL-13, tumor necrosis factor alpha (TNF-α), vascular endothelial growth factor (VEGF), monocyte chemoattractant protein-1 (MCP-1), macrophage inflammatory protein 1 alpha (MIP-1α), and interferon gamma-induced protein 10 (IP-10). The assay was performed using plasma samples collected from patients before hUC-MSC transplantation and every day after transplantation for 7 days. The plasma levels of each cytokine in relation to adjunctive hUC-MSC treatment were determined and compared to assess the effects of hUC-MSC transplantation on inflammatory cytokines. To measure cytokines and growth factors, 50 μL of plasma per sample was incubated with beads that could be resolved in the fluorescence channels of a flow cytometer. Cytokines and growth factors in each sample were captured using the corresponding beads. The cytokine/growth factor-captured beads were then mixed with phycoerythrin-conjugated detection antibodies to form sandwich complexes, according to the manufacturer’s instructions. Following incubation and washing, each plasma sample was diluted 1:3 with the sample diluent and analyzed by flow cytometry. The fluorescent signals were converted to concentrations (pg/mL) using a standard curve generated per assay.

### Demographics, clinical, and biological variables

Clinical data including age, sex, body mass index, smoking, comorbidities, initial onset symptoms, vital signs at initial presentation, long-term use of medications, COVID-19 vaccination status, laboratory data, and chest radiography were collected from all study patients. Arterial partial pressure of oxygen (PaO_2_) vs. fraction of inspired oxygen (FiO_2_), abridged as the PaO_2_/FiO_2_ ratio, was plotted as a line chart against the designated hospital days. We also calculated the change in the PaO_2_/FiO_2_ ratio (ΔPaO_2_/FiO_2_ ratio) between baseline (day 0) and day 7 following hUC-MSC infusion (hUC-MSC group) or day 7 after hospitalization (non-hUC-MSC group) in each patient with severe or critical disease to assess the treatment effects.

### Statistical analysis

Continuous data are reported as medians (interquartile range: 25th–75th percentile) and were analyzed using the Mann–Whitney *U*-test. Categorical variables are presented as n (%) and were compared using the chi-squared test or Fisher’s exact test. Repeated measures analysis of variance was applied to evaluate *p* for trends in changes in the PaO_2_/FiO_2_ ratio and cytokines. For cytokine analyses, the values of inflammatory biomarkers at all visits were plotted in line graphs. The mean values of inflammatory biomarkers at each visit were calculated and plotted on the same graph with black lines. The Mann–Kendall trend test was applied to determine any monotonous changes in the inflammatory biomarkers. Statistical analysis was performed using SPSS Statistics version 22 software (IBM^®^ SPSS^®^ Statistics, Chicago, United States).

## Results

**FIGURE 2:**
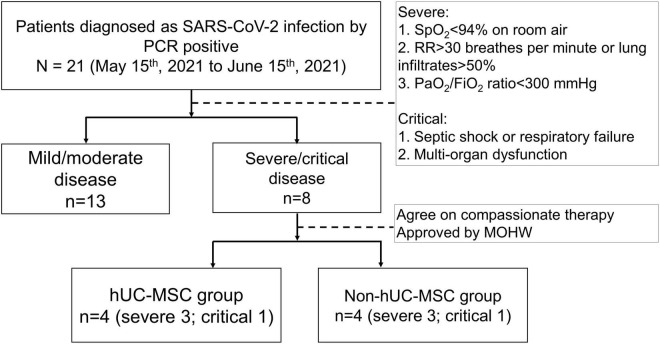
The flow diaphragm of the study. Hospitalized patients who were diagnosed with 2019 novel coronavirus disease (COVID-19) by a positive real-time polymerase chain reaction (RT-PCR) for SARS-CoV-2 from nasal swab specimens were enrolled in this retrospective case control study between May 15, 2021, and June 15, 2021. The disease severity of COVID-19 affected patients was classified as mild, moderate, severe and critical conditions according to the National Institutes of Health treatment guidelines. A total of 21 patients with RT-PCR-confirmed SARS-CoV-2 infection were enrolled, of whom 13 patients were classified as having a mild/moderate disease and 8 as a severe/critical disease. Patients with severe/critical disease were separated into hUC-MSC and non-hUC-MSC groups. SARS-CoV-2, severe acute respiratory syndrome coronavirus 2; PCR, polymerase chain reaction; SpO_2_, oxyhemoglobin saturation by pulse oximetry; RR, respiratory rate; PaO_2_/FiO_2_ ratio, arterial partial pressure of oxygen to fraction of inspired oxygen ratio; MOHW, Ministry of Health and Welfare; hUC-MSC, human umbilical cord mesenchymal stem cell.

Between May 15, 2021 and June 15, 2021, a total of 21 patients with RT-PCR-confirmed SARS-CoV-2 infection were enrolled, of whom 13 were classified as having mild or moderate disease and 8 as having severe or critical disease (Figure 2). Table 1 shows the demographic and clinical characteristics of study participants. Of the eight patients with severe or critical disease, the median age was older (59.5 [54-63.75] vs. 20 [19-53.5] years, *P* = 0.016), with a tendency toward male predominance (87.5% vs. 46.15%, *P* = 0.085) than in those with mild or moderate disease. There were no statistically significant differences in body mass index, smoking, comorbidities, symptoms at presentation, and body temperature between patients with severe or critical disease and those with mild or moderate disease. Notably, in patients with severe or critical disease, the pulse rate was lower (73.5 [70-79.5] vs. 91 [77.5-114], *P* = 0.025), respiratory rate was higher (25 [20-31] vs. 19 [18-20], *P* = 0.005), and oxyhemoglobin saturation was lower (96 [96–97.5] vs. 99 [96.5-99], *P* = 0.045) than in those with mild or moderate disease with supplemental oxygen.

**TABLE 1:** Demographics and baseline characteristics of patients.

	All patients (*N* = 21)	Mild/moderate disease (*N* = 13)	Severe/critical disease (*N* = 8)	*P*-value
Age	51.00 [19.00–62.00]	20.00 [19.00–53.50]	59.50 [54.00–63.75]	0.016
Male	13.00 (61.90)	6.00 (46.15)	7.00 (87.50)	0.085
Body mass index	23.40 [21.25–27.45]	22.80 [20.10–26.95]	24.95 [22.80–28.65]	0.238
Smoking	7 (33.33)	4 (30.77)	3 (37.50)	1.000
**Comorbidities**				
Diabetes mellitus	3.00 (14.29)	1.00 (7.69)	2.00 (25.00)	0.531
Hypertension	3.00 (14.29)	1.00 (7.69)	2.00 (25.00)	0.531
Hyperlipidemia	3.00 (14.29)	1.00 (7.69)	2.00 (25.00)	0.531
Coronary artery disease	3.00 (14.29)	2.00 (15.38)	1.00 (12.50)	1.000
Solid-organ malignancy*	2.00 (9.52)	1.00 (7.69)	1.00 (12.50)	1.000
Chronic obstructive pulmonary disease	0	0	0	-
Chronic kidney disease	0	0	0	-
Chronic liver disease**	0	0	0	-
**Symptoms**				
Fever***	10.00 (47.62)	5.00 (38.46)	5.00 (62.50)	0.387
Myalgia	2.00 (9.52)	1.00 (7.69)	1.00 (12.50)	1.000
Respiratory symptoms****	18.00 (85.71)	10.00 (76.92)	8.00 (100.00)	0.257
Gastrointestinal symptoms*****	6.00 (28.57)	1.00 (7.69)	1.00 (12.50)	0.146
Smell or taste dysfunction	1.00 (4.76)	1.00 (7.69)	0	1.000
**Vital signs**				
Body temperature (°C)	37.30 [37.10–37.80]	37.40 [37.25–37.85]	37.15 [36.58–37.28]	0.053
Pulse rate (/minute)	81.00 [73.00–107.50]	91.00 [77.50–114.00]	73.50 [70.00–79.50]	0.025
Respiratory rate (/minute)	20.00 [18.00–22.00]	19.00 [18.00–20.00]	25.00 [20.00–31.00]	0.005
SpO_2_ (%)	98.00 [96.00–99.00]	99.00 [96.50–99.00]	96.00 [96.00–97.50]	0.045
**Medications**				
ACEI/ARB	2.00 (9.52)	1.00 (7.69)	1.00 (12.50)	1.000
Other anti-hypertensive agents	0	0	0	-
Beta-blockers	0	0	0	-
Oral anti-diabetes agents	2.00 (9.52)	1.00 (7.69)	1.00 (12.50)	1.000
Lipid lowering agents	4.00 (19.04)	2.00 (15.38)	2.00 (25.00)	0.618
Corticosteroid	0	0	0	-
COVID-19 vaccination	0	0	0	-

Continuous variables are shown as median [IQR] and evaluated by the Mann–Whitney U-test, and category variables are shown as n (%) and analyzed by Fisher’s exact test. IQR, interquartile range; SpO_2_, oxyhemoglobin saturation by pulse oximetry; ACEI, angiotensin-converting enzyme inhibitors; ARB, angiotensin receptor blocker.

*Including prostate cancer.

**Including chronic hepatitis B, chronic hepatitis C, and liver cirrhosis.

***Defined central temperature equal or greater than 38.3°CC.

****Including headache, rhinorrhea, sore or itchy throat, cough, hemoptysis, and shortness of breath.

*****Poor appetite, nausea, vomiting, diarrhea, and abdominal pain.

Four out of eight patients with severe or critical disease received either one (*n* = 2) or two (*n* = 2) intravenous infusions of hUC-MSCs using a uniform cell dose of 1.0 × 10^8^. There were no significant differences in age, sex, body mass index, presenting symptoms, frequency of comorbidities, vital signs, or oxyhemoglobin saturation between hUC-MSC-treated and non-hUC-MSC-treated patients (Supplementary Table S1). There were no adverse events (17, 19) noticed during hUC-MSC infusion or within 24 h. Laboratory data including leukocyte count, leukocyte subgroups, hemoglobin level, platelet count, liver and renal functions, lactate dehydrogenase, creatine kinase, D-dimer, and electrolytes did not change significantly from baseline (day 0) through days 3 and 7 among patients with or without hUC-MSC transplantation (Table 2). Notably, the high-sensitivity C-reactive protein (hs-CRP) level showed a fast and significant decline over time in the hUC-MSC group (day 0: 7.25 [3.35–13.16]; day 3: 0.33 [0.10–2.49]; day 7: 0.05 [0.03–0.11], *P* for trend = 0.014) compared with the non-hUC-MSC group (day 0: 6.32 [0.63–11.73]; day 3: 2.18 [0.63–5.14]; day 7: 0.26 [0.06–0.98], *P* for trend = 0.196) (Table 2). Similarly, the monocyte distribution width (MDW) decreased significantly from 25.78 (5.31–28.96) on day 0 to 22.88 (16.51–24.50) on day 3 and then to 18.21 (15.53–18.65) on day 7 (*P* = 0.003) in patients receiving hUC-MSC transplantation, while the MDW did not differ significantly in non-hUC-MSC patients (day 0: 26.81 [23.51–33.43]; day 3: 23.60 [22.57–30.91]; day 7: 18.91 [17.96–24.71], *P* = 0.202) (Tables 2, 3).

**TABLE 2:** Serial laboratory data of the human umbilical cord mesenchymal stem cell (hUC-MSC) group.

	hUC-MSC group (*N* = 4)			*P*-value
		
	Day 0	Day 3	Day 7	
White blood cells (1000/μl)	4.45 [3.08–7.18]	7.75 [3.53–11.45]	6.25 [3.68–9.20]	0.086
Neutrophil (%)	71.00 [63.98–82.60]	80.65 [77.88–83.20]	70.30 [62.20–78.03]	0.315
Lymphocyte (%)	16.90 [8.40–25.70]	9.55 [7.68–12.10]	16.20 [2.73–25.10]	0.534
Monocyte (%)	9.25 [7.65–12.13]	7.65 [6.15–11.63]	9.75 [7.28–14.40]	0.073
Eosinophil (%)	0.15 [0.00–0.60]	0.70 [0.15–1.78]	1.20 [0.28–2.20]	0.312
Basophil (%)	0.35 [0.30–1.00]	0.20 [0.10–0.60]	0.50 [0.25–0.90]	0.515
Hemoglobin (g/dL)	14.25 [11.68–15.48]	13.90 [10.33–15.08]	13.35 [12.13–13.90]	0.600
Platelet (*1000/μl)	172.5 [94.75–252.50]	227.00 [117.00–312.25]	197.00 [132.25–294.75]	0.248
MDW	25.78 [5.31–28.96]	22.88 [16.51–24.50]	18.21 [15.53–18.65]	0.003
D-dimer (ng/ml)	824.80 [483.38–1141.93]	6378.80 [4615.70–19212.65]	3348.95 [2524.90–4345.88]	0.176
Creatinine (mg/dL)	0.90 [0.69–1.08]	0.84 [0.77–0.98]	0.74 [0.68–0.91]	0.406
GFR (ml/min/1.73 m^2^)	79.50 [62.00–115.75]	95.50 [63.25–100.00]	97.50 [75.50–118.75]	0.488
Total bilirubin (mg/dL)	0.43 [0.32–0.44]	0.94 [0.61–1.41]	0.68 [0.40–0.94]	0.037
AST (IU/L)	36.5 [23.75–107.75]	30.50 [24.25–33.75]	26.00 [18.25–33.00]	0.300
ALT (IU/L)	29.0 [18.5–66.5]	25.50 [19.00–44.00]	24.00 [18.00–44.25]	0.344
LDH (IU/L)	348 [236.75–476.50]	367.50 [249.25–569.00]	284.00 [249.25–464.25]	0.812
CPK (IU/L)	234.5 [116.50–777.00]	65.50 [43.25–129.00]	65.50 [29.25–135.50]	0.220
Troponin I (ng/mL)	0.01 [0.01–0.05]	0.03 [0.01–0.06]	0.01 [0.00–0.05]	0.315
hsCRP (mg/dL)	7.25 [3.35–13.16]	0.33 [0.10–2.49]	0.05 [0.03–0.11]	0.014
Albumin (g/dL)	3.70 [3.38–3.95]	3.40 [3.23–3.80]	3.45 [3.33–3.80]	0.285
Sodium (mmol/L)	134 [132.25–135.75]	136.00 [132.25–142.75]	139.00 [135.50–144.00]	0.068
Potassium (mmol/L)	4.5 [3.85–4.85]	4.35[4.13–4.58]	4.30 [3.80–4.65]	0.883

Continuous variables are shown as median [IQR] and evaluated by the Mann–Whitney U-test, and category variables are shown as n (%) and analyzed by Fisher’s exact test. MDW, monocyte distribution width; GFR, glomerular filtration rate; AST, aspartate aminotransferase; ALT, aminotransferase; LDH, lactate dehydrogenase; CPK, creatine kinase; hsCRP, high-sensitivity C-reactive protein.

**TABLE 3:** Serial laboratory data of the non-human umbilical cord mesenchymal stem cell (non-hUC-MSC) group.

	Non-hUC-MSC group (*N* = 4)			*P*-value
		
	Day 0	Day 3	Day 7	
White blood cells (1000/μl)	7.25 [5.15–9.65]	6.05 [5.15–7.70]	10.15 [6.95–12.68]	0.087
Neutrophil (%)	77.45 [67.48–83.90]	68.95 [61.23–81.48]	59.25 [14.15–77.50]	0.383
Lymphocyte (%)	10.80 [8.23–15.10]	19.15 [8.85–24.95]	15.75 [1.20–29.55]	0.598
Monocyte (%)	11.60 [7.80–14.50]	4.60 [0.00–9.20]	10.00 [8.85–11.90]	0.076
Eosinophil (%)	0.00 [0.00–2.03]	0.10 [0.03–0.33]	0.55 [0.00–1.48]	0.710
Basophil (%)	0.20 [0.03–0.90]	0.35 [0.23–0.40]	0.50 [0.15–0.85]	0.821
Hemoglobin (g/dL)	14.95 [13.98–15.25]	14.85 [14.50–14.90]	15.10 [14.08–15.75]	0.762
Platelet (*1000/μl)	240.00 [200.50–278.75]	246.50 [46.50–346.00]	271.00 [147.50–359.25]	0.710
MDW	26.81 [23.51–33.43]	23.60 [22.57–30.91]	18.91 [17.96–24.71]	0.202
D-dimer (ng/ml)	7069.30 [917.45–12956.78]	6841.50 [1240.23–40656.95]	3095.60 [1061.08–5694.43]	0.334
Creatinine (mg/dL)	0.86 [0.69–1.01]	0.79 [0.65–0.92]	0.78 [0.75–0.83]	0.597
GFR (ml/min/1.73 m^2^)	91.00 [76.50–119.75]	102.50 [83.75–127.25]	103.00 [94.50–107.00]	0.728
Total bilirubin (mg/dL)	0.52 [0.43–0.83]	0.68 [0.50–0.87]	0.92 [0.60–1.21]	0.093
AST (IU/L)	32.50 [25.50–44.75]	28.50 [24.25–40.25]	35.50 [26.00–44.25]	0.820
ALT (IU/L)	39.50 [18.50–65.75]	39.00 [28.25–40.25]	47.50 [24.25–60.25]	0.838
LDH (IU/L)	473.50 [254.00–608.25]	356.00 [284.25–427.00]	265.50 [241.75–405.50]	0.197
CPK (IU/L)	185.50 [44.25–334.25]	52.00 [22.50–535.25]	52.00 [40.00–137.50]	0.529
Troponin I (ng/mL)	0.01 [0.01–0.03]	0.02 [0.01–0.09]	0.01 [0.01–0.02]	0.357
hsCRP (mg/dL)	6.32 [0.63–11.73]	2.18 [0.63–5.14]	0.26 [0.06–0.98]	0.196
Albumin (g/dL)	3.30 [3.00–4.13]	3.30 [2.93–3.75]	3.40 [3.30–3.58]	0.732
Sodium (mmol/L)	140.00 [135.25–141.00]	141.50 [138.00–145.75]	140.00 [137.75–143.00]	0.280
Potassium (mmol/L)	4.40 [3.75–4.98]	4.35 [3.98–4.73]	4.20 [4.13–4.28]	0.819

Continuous variables are shown as median [IQR] and evaluated by the Mann–Whitney U-test, and category variables are shown as n (%) and analyzed by Fisher’s exact test. MDW, monocyte distribution width; GFR, glomerular filtration rate; AST, aspartate aminotransferase; ALT, aminotransferase; LDH, lactate dehydrogenase; CPK, creatine kinase; hsCRP, high-sensitivity C-reactive protein.

**FIGURE 3:**
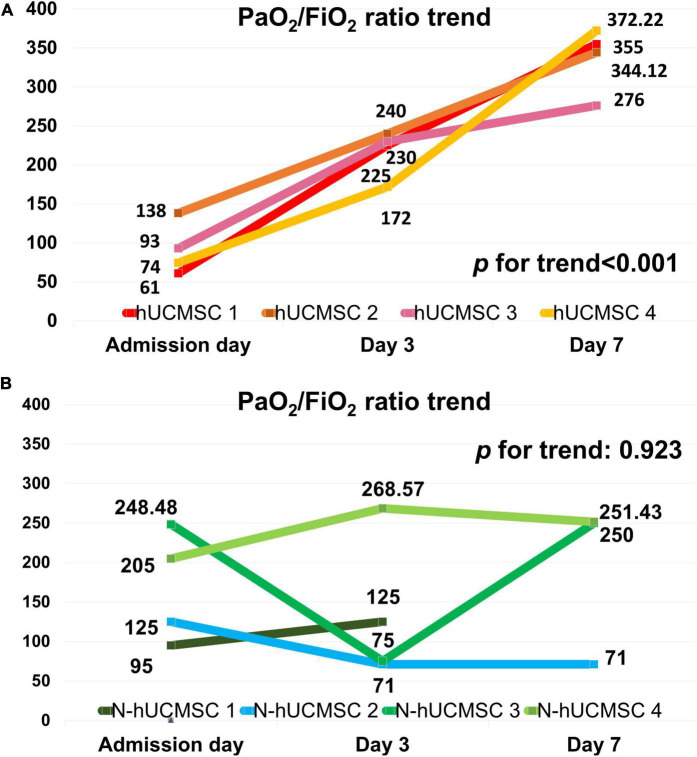
PaO_2_/FiO_2_ ratio trends on hUC-MSC and non-hUC-MSC patients. **(A)** PaO_2_/FiO_2_ ratio trend on hUC-MSC patients. In hUC-MSC group, data on the day of admission, post-hUC-MSC day 3, and day 7 were recorded. **(B)** PaO_2_/FiO_2_ ratio trend on non-hUC-MSC patients. In the non-hUC-MSC group, the PaO_2_/FiO_2_ ratio on the day of admission, day 3, and day 7 of hospitalization are listed. In non-hUC-MSC case 1, no further arterial gas examination needed on day 7 was judged by the primary care physician because of obvious clinical improvements in this patient. Abbreviations as in Figure 2.

**FIGURE 4:**
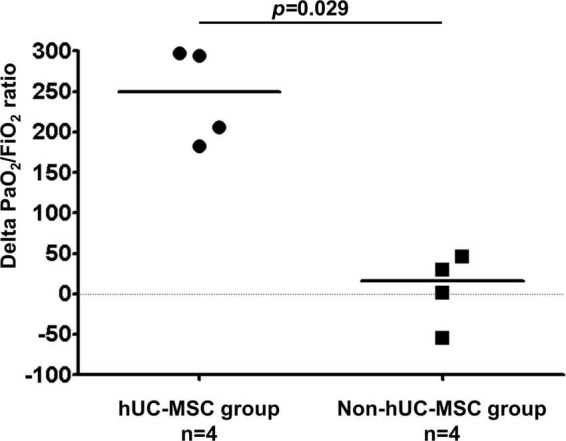
Delta PaO_2_/FiO_2_ ratio difference on hUC-MSC and non-hUC-MSC patients. The delta PaO_2_/FiO_2_ ratio which was the difference of PaO_2_/FiO_2_ ratio between the admission day and day 7 of hospitalization also showed a larger increment in the hUC-MSC treated patients than in non-hUC-MSC patients (250.06 [188.78–296.90] vs. 15.76 [–40.12–42.32], *P* = 0.029). Abbreviations as in Figure 2.

**FIGURE 5:**
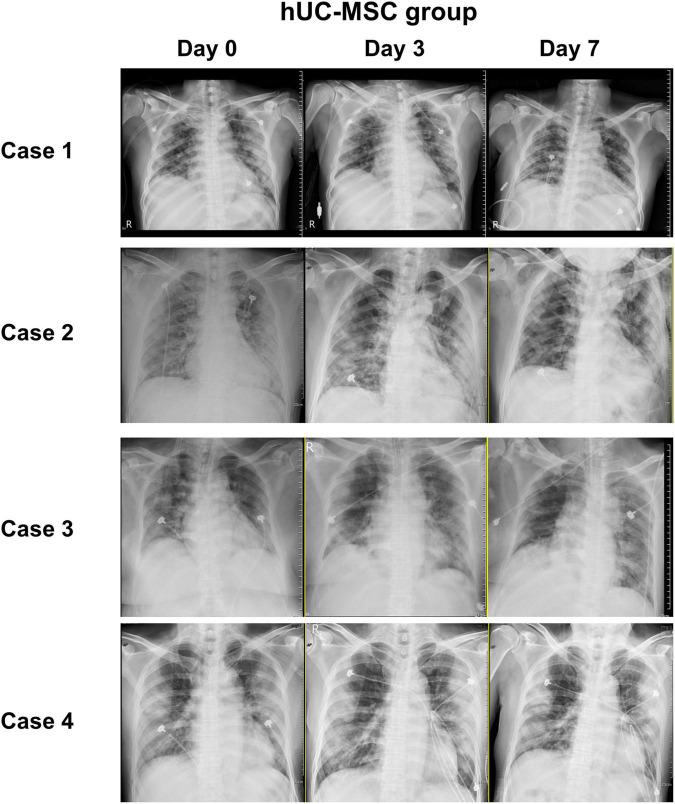
Chest image records of hUC-MSC patients. Serial chest X-ray films obtained on admission day, post-hUC-MSC day 3, and post-hUC-MSC day 7 showed progressive improvements on pulmonary infiltrations in all patients receiving hUC-MSC infusions. Abbreviations as in Figure 2.

Figure 3 shows that the median PaO_2_/FiO_2_ ratio increased from 83.68 [64.34–126.75] on day 0 to 227.50 [185.25–237.50] on post-hUC-MSC day 3 and to 349.56 [293.03–367.92] on hUC-MSC day 7 (*P* for trend <0.001) in patients receiving hUC-MSC, while the change in PaO_2_/FiO_2_ ratio was not significant in non-hUC-MSC patients (day 0: 165.00 [102.50–237.61]; day 3: 100.00 [72.00–232.68]; day 7: 250.00 [71.00–251.43], *P* for trend = 0.923). The ΔPaO_2_/FiO_2_ ratio also showed a larger increment in hUC-MSC-treated patients than in non-hUC-MSC patients (250.06 [188.78–296.90] vs. 15.76 [−40.12–42.32], *P* = 0.029) (Figure 4). In concordance with the changes in the PaO_2_/FiO_2_ ratio, serial chest X-ray films showed progressive improvements in pulmonary infiltration in all patients receiving hUC-MSC infusion (Figure 5). Three and four patients, with or without hUC-MSC treatment, respectively, were discharged from the hospital uneventfully with similar hospital stays. One patient in the hUC-MSC group showed an initial improvement in pulmonary oxygenation after hUC-MSC infusion (Figure 5) but was unfortunately complicated by central vein cannulation procedure-related pneumothorax, resulting in prolonged intubation and subsequent bacterial superinfection and multi-organ failure before expiration.

**FIGURE 6:**
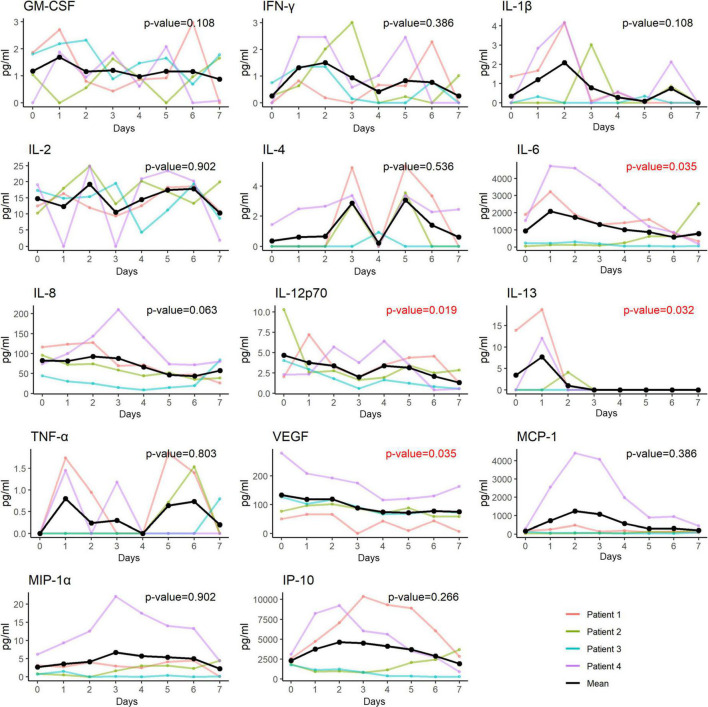
Changes of plasma levels of cytokines and chemokines in patients receiving hUC-MSC intervention. The plasma levels of 4 out of the 14 inflammatory cytokines including IL-6, IL-12p70, IL-13 and VEGF had a significant decrease in trend after receiving the hUC-MSC transplantation. GM-CSF, granulocyte-macrophage colony-stimulating factor; IFN, interferon; IL, interleukin; IP, interferon-gamma-inducible protein; MCP, monocyte chemoattractant protein; MIP, macrophage inflammatory protein; TNF, tumor necrosis factor; VEGF, vascular endothelial growth factor.

In accordance with the changes in both hs-CRP and MDW, the plasma levels of 4 of the 14 inflammatory cytokines, including IL-6, IL-12p70, IL-13, and VEGF, significantly decreased after hUC-MSC transplantation (Figure 6).

## Discussion

To our knowledge, the current study is the first to demonstrate the beneficial effects of allogenic hUC-MSC transplantation on pulmonary oxygenation and anti-inflammation, in addition to standard triple therapy with remdesivir, dexamethasone, and tocilizumab in patients with severe or critical COVID-19. The principal findings of the current study include: (1) compared to patients with mild or moderate COVID-19, patients with severe or critical infection were older with a lower pulse rate on presentation; (2) hUC-MSC transplantation resulted in a considerably higher improvement in the ΔPaO_2_/FiO_2_ ratio within the first 7 days of hospitalization, compared with those who did not receive hUC-MSC treatment; and (3) significant reductions in both MDW and hs-CRP along with a consistently decreasing trend of multiple cytokines including IL-6, IL-12p70, IL-13, and VEGF, attesting to the immunomodulatory effects of hUC-MSC treatment in these patients.

### Characteristics of severe or critical COVID-19 infection

Since mid-May 2021, the COVID-19 pandemic has been predominantly caused by the prevalence of the World Health Organization-labeled alpha (B.1.1.7 linage) SARS-CoV-2 infection, as reported by the Taiwan Centers for Disease Control (20). Similar to previous studies, patients with severe or critical disease were older than those with mild or moderate disease, indicating that older individuals are prone to severe or critical clinical courses (22, 23). In previous studies, dysgeusia and anosmia have been reported as being part of the clinical manifestations of COVID-19, with pooled prevalence rates of 41.0% and 38.2%, respectively (24, 25). We found that smell and taste dysfunction were relatively uncommon (4.76%) among all study patients during the alpha variant SARS-CoV-2 pandemic, irrespective of disease severity. Typically, patients with severe COVID-19 pneumonia exhibit hypoxemia and an increased heart rate as the initial manifestations (26). In contrast, relative bradycardia, defined as a pulse rate increment of less than 18 beats per minute at each Celsius degree elevation of body temperature (27), has been observed in a subset of patients with COVID-19 (28). Similarly, relative bradycardia was also found in our patients with severe or critical disease, where older age and male sex may be associated with these paradoxical reactions (28). The hypothetical mechanisms underlying relative bradycardia in patients with COVID-19 might be linked to the activation of inflammatory cytokines (27), which in turn leads to elevated hs-CRP levels in patients with severe or critical COVID-19 infection.

### Allogenic human umbilical cord mesenchymal stem cell transplantation on top of standard COVID-19 therapy

A 1 year after the COVID-19 pandemic, a number of treatment options have been established, of which remdesivir, dexamethasone, and tocilizumab are commonly used for hospitalized patients who require supplemental oxygen (2). Recently, several clinical trials or case studies using MSCs as an adjunctive therapy have shown beneficial effects in attenuating the cytokine storm provoked by COVID-19 owing to their potent immunomodulatory capability (16, 17, 29, 30). However, there have been no studies exploring the therapeutic efficacy of MSCs in addition to the standard triple regimens mentioned above for every individual patient with severe or critical COVID-19 infection. Our study is the first to demonstrate that adjunctive transplantation of allogenic hUC-MSCs improved the PaO_2_/FiO_2_ and ΔPaO_2_/FiO_2_ ratios under background therapy with remdesivir, dexamethasone, and tocilizumab in patients with severe or critical COVID-19 pneumonia compared to their counterparts with no hUC-MSC treatment. The improvements in the PaO_2_/FiO_2_ ratio paralleled the relief of respiratory distress, manifested by lower respiratory rates and pulmonary infiltrations on chest radiographs following hUC-MSC transplantation.

The underlying mechanisms responsible for improving the resolution of acute ARDS by MSCs transplantation have been attributed to their anti-apoptotic and anti-inflammatory effects on host cells, which attenuate alveolar epithelial permeability and enhance the phagocytic activity of mononuclear cells, thus leading to an increase in alveolar fluid clearance (31, 32). MSCs are capable of enhancing epithelial integrity through transportation of healthy mitochondria to epithelial cells to reduce apoptosis and oxidative damage (33, 34). Additionally, TNF stimulated gene-6, a potent anti-inflammatory protein secreted by MSCs, may contribute to reducing cell counts and inflammatory cytokine in bronchoalveolar lavage fluid, resulting in resolution of lung fibrosis (35).

It is worth noting that in addition to the rapid decline in the hs-CRP levels in hUC-MSC-treated patients, the MDW level also displayed a consistent decreasing pattern, which was not observed in patients who did not receive hUC-MSC treatment. MDW is considered a new biomarker for inflammation and sepsis (36), which we found for the first time to be modified by hUC-MSC treatment, indicating that hUC-MSCs may help prevent secondary bacterial infection. All these lines of evidence attest to the powerful immunomodulatory ability of allogeneic hUC-MSCs in attenuating pulmonary and systemic inflammation in patients with severe or critical COVID-19. Nonetheless, one patient who received delayed (6 days after hospitalization) hUC-MSC infusions also exhibited improvement in oxygenation status and pulmonary infiltration but unfortunately died of procedure-related pneumothorax and multiple organ failure. Whether the early use of hUC-MSCs may exert more beneficial effects remains unknown and requires further study. Among the four patients with severe or critical disease, two received only one dose of intravenous infusions of hUC-MSCs at a uniform cell dose of 1.0 × 10^8^. Both patients exhibited a favorable response and were discharged uneventfully, similar to those receiving two doses of UC-MSC treatment, as reported previously. Thus, the optimal cell dosage should be determined in future studies.

### Inflammatory cytokines, monocyte distribution width, and high-sensitivity c-reactive protein

The pathophysiological mechanisms underlying SARS-CoV-2 infection involve the activation of a cytokine storm in the lung with recruitment of a number of cytokines, chemokines, and growth factors, such as IL-1β, IL-2, IL-6, IL-8, G-CSF, IP-10, MCP-1, MIP-1α, MIP-1β, IFN-γ, and TNF-α, followed by lung edema, impairment of air exchange, and acute respiratory distress syndrome, which in turn leads to secondary infection, multiple organ failure, and eventually death (3, 37, 38). MSC treatment can suppress the overactivation of the immune system and promote lung repair to improve the pulmonary microenvironment and prevent fibrosis (14). Zhu et al. (39) further explored the underlying mechanisms of the immunomodulatory functions of MSCs using single-cell RNA sequencing analysis of the peripheral blood. They demonstrated that mobilization of a subpopulation of VNN2^+^ hematopoietic stem/progenitor-like cells expressing CSF3R and PTPRE occurred after MSC infusion. In addition, upregulation of genes encoding chemotaxis factors (CX3CR1 and L-selectin) occurs in various immune cells. MSC infusion also favorably regulated B cell subpopulations and promoted the expression of costimulatory CD28 in T cells. Together, these pathways contribute to clinical outcome improvements in patients with COVID-19 via maintenance of immune homeostasis. In our patients, some cytokines (including IL-6, IL-13, IL-12p70, and VEGF) were significantly decreased compared to others (GM-CSF, IFN-γ, IL-1β, IL-2, IL-4, IL-8, TNF-α, MCP-1, MIP-1α, and IP-10), which appears to be slightly different from the findings of previous studies (14, 17, 21, 29). The possible reasons for the discrepancy in changes in the cytokine profile following MSC transplantation may be attributable to the relatively lower baseline plasma concentrations of some cytokines, such as TNF-α (<3 pg/mL) in our patients, which may render it difficult to see the difference (29). It is also possible that differences in treatment might have influenced the expression levels of these pro-inflammatory cytokines, since all our patients received standard triple therapy with remdesivir, dexamethasone, and tocilizumab; however, not all enrolled patients in previous studies received standard triple therapy (14, 29). Nevertheless, the significant decline in certain inflammatory cytokines in our patients shortly after hUC-MSC infusion attests to the beneficial immunomodulatory role of MSCs in severe or critical patients with COVID-19.

Another frequently discussed issue is multisystem inflammatory syndrome in children (MIS-C), characterized by inflammatory reactions, fever, and multi-organ dysfunction after SARS-CoV-2 infection. The mechanism of MIS-C is associated with a hyperinflammatory status or cytokine storm, which involves multiple inflammatory cytokines such as IL-1 and IL-6 (40). To date, there is very limited evidence regarding the clinical application of MSCs in MIS-C. Given that MSCs exert various counteracting mechanisms, including the conversion of Th17 cells to anti-inflammatory FOXP3 T-regulatory cells and the promotion of inflammatory M1 macrophages to anti-inflammatory M2 macrophages against the pathological consequences associated with MIS-C, this therapy holds promise as an effective treatment for patients with MIS-C (41–44).

Several strategies, such as vaccines, have been developed to eradicate this pandemic, and there are numerous validated vaccine products comprising different mechanisms, including messenger RNA (mRNA) with nanoparticles, viral vectors, and protein subunits, to generate an adequate immune response (45). However, these vaccines have an average development time of 1–2 years (46) and may not meet the emergence of novel variants of SARS-CoV-2. In early 2022, a new variant of SARS-CoV-2, Omicron, overwhelmed Hong Kong and led to an overall 129 deaths per million population of fully vaccinated individuals with at least two doses of validated vaccines (47). Another study focusing on the protection of vaccine showed an average of 90% effectiveness against intensive care unit admission (48). Despite prevention policies such as vaccination, morbidity and mortality still remain, which highlights the importance of multimodal treatment strategies. Our study showed that, in addition to standard triple therapy, infusion of hUC-MSCs was useful in patients with severe or critical severity of COVID-19. We believe that this approach should be included as an effective intervention for intractable SARS-CoV-2 infections.

Monocyte distribution width, a cytomorphological indicator that correlates with inflammatory markers, including CRP, fibrinogen, and ferritin, has recently emerged as a valuable biomarker for severe COVID-19 and sepsis (49). Similar to sepsis, monocytes and macrophages play an important role in triggering life-threatening hyperinflammation in patients with severe COVID-19. Previous studies have shown that high MDW values not only correlate with COVID-19 severity but are also prognostically associated with fatal outcomes in these patients (49). Herein, we demonstrated for the first time that the median MDW value (25.78), which was above the optimal diagnostic threshold (23.5) for sepsis prediction (50), was significantly reduced within 7 days following adjunctive hUC-MSC transplantation in patients with severe or critical COVID-19. A parallel reduction in hs-CRP level was also observed in these patients. These findings also substantiate the previous notion that MSCs might exert their antimicrobial effects through secretion of antimicrobial peptides or expression of bactericidal molecules, such as indoleamine 2,3-dioxygenase and IL-17 (51–53).

### Limitations

Our study had several limitations. First, the number of cases of severe or critical COVID-19 infection was small, which prompts the need for larger-scale studies to confirm the effectiveness and safety of hUC-MSC administration as an adjunctive therapy in addition to standard triple therapy with remdesivir, dexamethasone, and tocilizumab. Second, this was a retrospective case-control study of the compassionate use of hUC-MSCs; thus, potential bias or confounders might exist when deriving comparative results. In addition, the lack of a day 7 PaO_2_/FiO_2_ ratio in one of the patients from the non-MSC group could potentially confound the data interpretation. To minimize the potential bias related to the small sample size and a highly skewed statistical distribution, we used non-parametric statistics, such as the Chi-square test, Fisher’s exact test, Mann–Whitney *U*-test, and Mann–Kendall trend test, for analyses. Indeed, the number of patients with severe or critical COVID-19 enrolled in our study was small, which prompts the need for larger-scale studies to confirm the effectiveness and safety of hUC-MSC administration as an adjunctive therapy in addition to standard triple therapy with remdesivir, dexamethasone, and tocilizumab in the future. Third, we only examined inflammatory cytokines in hUC-MSC-treated patients but not in non-hUC-MSC patients. Therefore, the beneficial effects of hUC-MSC transplantation on mitigating inflammatory cytokine levels need to be confirmed by performing the same examination in control patients in randomized studies. Lastly, we only performed chest X-rays to monitor pneumonia progression, while most studies focused on patterns of COVID-19 using follow-up chest computed tomography (54, 55), which is considered a better tool to assess changes in chest imaging.

## Conclusion

Transplantation of allogenic hUC-MSCs as an adjunctive therapy in addition to a standard triple regimen of remdesivir, dexamethasone, and tocilizumab further improved pulmonary oxygenation status in patients with severe or critical COVID-19. The beneficial effects of hUC-MSC treatment were presumably mediated by the mitigation of inflammatory cytokines, characterized by the reduction in both hs-CRP and MDW.

## Data availability statement

The original contributions presented in this study are included in the article/Supplementary material, further inquiries can be directed to the corresponding author/s.

## Ethics statement

The studies involving human participants were reviewed and approved by Ethical Committee of the China Medical University Hospital (CMUH-110-REC1-123). The patients/participants provided their written informed consent to participate in this study.

## Author contributions

C-HaC, K-CC, W-CS, and L-BJ conceived the study concept and design. C-HaC, Y-NL, M-WH, M-YC, W-HH, C-HuC, P-CL, C-YC, M-CL, and NT performed the acquisition of data. C-HaC, K-CC, Y-NL, M-YW, W-CS, and L-BJ contributed to the first drafting of the manuscript. C-HaC, K-CC, and M-YW carried out the statistical analysis. K-CC obtained the funding. K-CC, W-CS, D-YC, and L-BJ supervised the study. K-CC, W-HS, and L-BJ contributed to the data access and responsibility and had full access to all the data in the study and take responsibility for the integrity of the data and the accuracy of the data analysis. All authors contributed to the analysis and interpretation of data and critically revised the manuscript for important intellectual content.

## Abbreviations

COVID-19: coronavirus disease 2019
FiO_2_: fraction of inspired oxygen
GM-CSF: granulocyte-macrophage colony-stimulating factor
HFNC: high-flow nasal cannula
hsCRP: high-sensitivity C-reactive protein
hUC-MSCs: human umbilical cord MSCs
IFN- γ: interferon gamma
IL: interleukin
IP-10: interferon gamma-induced protein 10
MCP-1: monocyte chemoattractant protein-1
MDW: monocyte distribution width
MIP-1 α: macrophage inflammatory protein 1 alpha
MSCs: mesenchymal stem cells
PaO_2_: arterial oxygen partial pressure
RT-PCR: real-time polymerase chain reaction
TNF- α: tumor necrosis factor alpha
VEGF: vascular endothelial growth factor
MIS-C: multisystem inflammatory syndrome in children
SARS: severe acute respiratory syndrome.
