# Supplementary material for: Mesenchymal stem cell therapy on top of triple therapy with remdesivir, dexamethasone, and tocilizumab improves PaO2/FiO2 in severe COVID-19 pneumonia
Source: Front Med (Lausanne). 2022 Sep 23;9:1001979. doi: 10.3389/fmed.2022.1001979 (PMC9537613; doi:10.3389/fmed.2022.1001979)
Supplement: Supplementary file 1 [file Presentation_1.pdf]

Mesenchymal Stem Cell Therapy on Top of Triple Therapy with Remdesivir, Dexamethasone, and Tocilizumab Improves PaO_2/FiO_2 in Severe COVID-19 Pneumonia

Chih-Hao Chen, MD[1]†; Kuan-Cheng Chang, MD, PhD [2,3]†; Yen-Nien Lin, MD, PhD[2,3]; Mao-Wang Ho, MD[1]; Meng-Yu Cheng, MD[1]; Wen-Hsin Shih, MD[1]; Chia-Huei Chou, MD[1]; Po-Chang Lin, MD[1]; Chih-Yu Chi, MD[1]; Min-Chi Lu[1,4]; Ni Tien[5]; Mei-Yao Wu[6,7]; Shih-Sheng Chang, MD, PhD[2]; Wu-Huei Hsu, MD[3,8]; Woei-Cheang Shyu, MD, PhD[3,9,10,11]; Der-Yang Cho, MD, PhD[3,12,13]; Long-Bin Jeng, MD[3,14]

†These authors contributed equally to this work.

[1]Division of Infectious Diseases, Department of Internal Medicine, China Medical University Hospital, Taichung 40447, Taiwan

[2]Division of Cardiovascular Medicine, Department of Internal Medicine, China Medical University Hospital, Taichung 40447, Taiwan

[3]School of Medicine, China Medical University, Taichung 40402, Taiwan

[4]Department of Microbiology and Immunology, School of Medicine, China Medical University, Taichung 40402, Taiwan

[5]Department of Laboratory Medicine, China Medical University Hospital, Taichung, Taiwan

[6]School of Post-Baccalaureate Chinese Medicine, China Medical University, Taichung 40402, Taiwan

[7]Department of Chinese Medicine, China Medical University Hospital, Taichung 40447, Taiwan

[8]Division of Pulmonary Medicine, Department of Internal Medicine, China Medical

University Hospital, Taichung 40447, Taiwan

[9]Translational Medicine Research Center, China Medical University Hospital, Taichung 40447, Taiwan

[10]Department of Neurology, China Medical University Hospital, Taichung 40447, Taiwan

[11]Graduate Institute of BioMedical Science, China Medical University, Taichung 40402, Taiwan

[12]Stroke Center, China Medical University Hospital, Taichung 40447, Taiwan

[13]Department of Neurosurgery, China Medical University Hospital, Taichung 40447, Taiwan

[14]Organ Transplantation Center, China Medical University Hospital, Taichung 40447, Taiwan

Supplementary table S1: Demographics of patients in MSC and non-MSC groups

Variables	MSC group (N = 4)	Non-MSC group (N = 4)	p value
Age	61.50 [53.00–71.50]	58.50 [54.00–62.25]	0.686
Male sex	3.00 (75.00)	4.00 (100.00)	1.000
Body mass index	24.25 [21.33–29.43]	25.95 [23.55–28.65]	0.686
Smoking	1.00 (25%)	2.00 (50%)	1.000
Comorbidities			
Diabetes mellitus	1.00 (25%)	1 (25%)	1.000
Hypertension	1.00 (25%)	1 (25.00)	1.000
Hyperlipidemia	1.00 (25%)	1.00 (25%)	1.000
Coronary artery disease	0	1.00 (25%)	1.000
Solid-organ malignancy*	1.00 (25%)	0	1.000
Chronic obstructive pulmonary disease	0	0	-
Chronic kidney disease	0	0	-
Chronic liver disease**	0	0	-
Symptoms			
Fever***	2.00 (50%)	3 (75%)	1.000
Myalgia	1.00 (25%)	0	1.000
Respiratory symptoms****	4.00 (100%)	4 (100%)	-

Gastrointestinal symptoms*****	1.00 (25%)	0	1.000
Smell or taste dysfunction	0	0	-
Vital signs			
Body temperature (℃)	37.25 [37.13–39.48]	36.75 [36.25–37.18]	0.114
Pulse rate (/minute)	73.50 [69.25–87.50]	74.00 [70.00–79.50]	1.000
Respiratory rate (/minute)	27.00 [20.00–31.00]	22.00 [20.00–32.25]	0.886
SpO2 (%)	96.00 [96.00–97.50]	96.00 [95.25–98.25]	0.886
Medications			
ACEI/ARB	1.00 (25%)	0	1.000
Other anti-hypertensive agents	0	0	-
Beta-blockers	0	0	-
Oral anti-diabetes agents	1.00 (25%)	0	1.000
Lipid lowering agents	1.00 (25%)	1.00 (25%)	1.000
Corticosteroid	0	0	-
COVID-19 vaccination	0	0	-

Continuous variables are shown as median [IQR] and evaluated by the Mann-Whitney U test, and category variables are shown as n (%) and analyzed by Fisher's exact test.

MSC, mesenchymal stem cell; IQR, interquartile range; SpO_2, oxyhemoglobin saturation by pulse oximetry; ACEI, angiotensin-converting enzyme inhibitors; ARB, angiotensin receptor blocker.

*including prostate cancer

**including chronic hepatitis B, chronic hepatitis C, liver cirrhosis

***defined central temperature equal or greater than 38.3 ℃

****including headache, rhinorrhea. sore or itching throat, cough, hemoptysis, shortness of breath

*****poor appetite, nausea, vomiting, diarrhea, abdominal pain
